# Supplementary material for: Leukemia Incidence by Occupation and Industry: A Cohort Study of 2.3 Million Workers from Ontario, Canada
Source: Int J Environ Res Public Health. 2024 Jul 27;21(8):981. doi: 10.3390/ijerph21080981 (PMC11353350; doi:10.3390/ijerph21080981)
Supplement: Supplementary file 1 [file ijerph-21-00981-s001.zip › ijerph-3092442-supplementary.pdf]

## Supplementary Material

**Table S1.** Hazard ratios and 95% confidence intervals for leukemia by division and major level industry groups, stratified by sex.

| Industry Group (SIC Code) | Leukemia [a] | | | |
|---|---|---|---|---|
| | Females (n = 1,810 Cases) | | Males (n = 5,464 Cases) | |
| | Cases (Workers) [b] | HR (95% CI) [c] | Cases (Workers) [b] | HR (95% CI) [c] |
| Agriculture (1) | 19 (9557) | 1.14 (0.73-1.79) | 67 (28,371) | 0.90 (0.71-1.15) |
| Forestry, Fishing and Trapping (2/3) | <6 (499) | -- | 41 (10,240) | 0.98 (0.72-1.33) |
| *- Forestry* | <6 (459) | -- | 41 (9823) | 1.02 (0.75-1.38) |
| Mines, Quarries, and Oil Wells (4) | <6 (663) | -- | 145 (24,235) | 1.27 (1.08-1.50) |
| *- Metal Mines* | <6 (279) | -- | 107 (13,987) | 1.39 (1.15-1.68) |
| *- Non-Metal Mines* | <6 (158) | -- | 11 (1908) | 1.25 (0.69-2.26) |
| *- Quarries and Sandpits* | <6 (125) | -- | 14 (4101) | 1.00 (0.59-1.69) |
| *- Services Incidental to Mining* | <6 (102) | -- | 16 (4671) | 0.99 (0.61-1.62) |
| Manufacturing Industries (5) | 428 (172,174) | 0.98 (0.88-1.09) | 2064 (544,760) | 0.99 (0.93-1.04) |
| *- Food and Beverage* | 78 (29,220) | 1.10 (0.87-1.38) | 256 (63,977) | 1.10 (0.97-1.25) |
| *- Rubber and Plastics Products* | 43 (18,628) | 1.01 (0.75-1.37) | 92 (36,575) | 0.79 (0.64-0.97) |
| *- Leather and Allied Industries* | 8 (3522) | 0.81 (0.40-1.62) | 8 (2222) | 0.88 (0.44-1.75) |
| *- Textile* | 29 (9091) | 1.15 (0.79-1.65) | 31 (10,676) | 0.79 (0.56-1.13) |
| *- Clothing* | 23 (7504) | 0.90 (0.59-1.35) | 7 (2795) | 0.60 (0.28-1.25) |
| *- Wood* | 10 (4442) | 1.12 (0.60-2.09) | 79 (35,642) | 0.76 (0.61-0.95) |
| *- Furniture and Fixtures* | 26 (6581) | 1.52 (1.03-2.23) | 77 (27,045) | 0.81 (0.65-1.02) |
| *- Paper and Allied Industries* | 15 (5310) | 1.06 (0.64-1.77) | 81 (20,509) | 0.94 (0.76-1.18) |
| *- Printing, Publishing, and Allied Industries* | 20 (9006) | 0.91 (0.59-1.42) | 66 (17,691) | 1.11 (0.87-1.41) |
| *- Primary Metal Industries* | <6 (2589) | -- | 198 (40,148) | 1.05 (0.91-1.21) |
| *- Metal Fabricating* | 44 (19,821) | 0.96 (0.71-1.30) | 353 (112,963) | 0.94 (0.84-1.05) |
| *- Machinery* | <6 (294) | -- | 29 (6236) | 1.00 (0.69-1.44) |
| *- Electrical Product* | 41 (15,654) | 0.91 (0.67-1.24) | 79 (23,246) | 0.84 (0.67-1.05) |
| *- Transportation Equipment* | 66 (25,183) | 1.21 (0.94-1.55) | 529 (106,691) | 1.20 (1.10-1.32) |
| *- Non-Metallic Mineral Products* | 8 (2764) | 1.06 (0.53-2.12) | 81 (23,025) | 0.93 (0.75-1.16) |
| *- Chemical and Chemical Products* | 10 (6623) | 0.60 (0.32-1.11) | 52 (16,100) | 0.90 (0.68-1.18) |
| *- Miscellaneous Manufacturing* | 21 (11,956) | 0.69 (0.45-1.06) | 179 (45,854) | 1.03 (0.89-1.20) |
| Construction (6) | 21 (10,942) | 1.16 (0.76-1.79) | 712 (216,470) | 1.03 (0.95-1.12) |
| *- General Contractors* | 11 (4726) | 1.24 (0.68-2.24) | 259 (72,204) | 1.01 (0.89-1.14) |
| *- Special-Trade Contractors* | 10 (6256) | 1.08 (0.58-2.02) | 483 (153,392) | 1.04 (0.95-1.14) |
| Transportation, Communication and Other Utilities (7) | 73 (37,710) | 1.09 (0.86-1.38) | 681 (176,828) | 1.11 (1.03-1.21) |
| *- Transportation* | 38 (21,496) | 1.11 (0.81-1.54) | 465 (125,934) | 1.09 (0.99-1.20) |
| *- Communication* | 26 (12,220) | 0.99 (0.67-1.46) | 91 (22,058) | 1.06 (0.86-1.31) |
| *- Electric, Power, Gas, and Water Utilities* | <6 (2425) | -- | 112 (21,677) | 1.33 (1.10-1.61) |
| *- Storage* | 6 (1745) | 2.85 (1.28-6.37) | 28 (9896) | 1.11 (0.76-1.61) |
| Wholesale and Retail Trade (8) | 324 (154,321) | 1.12 (0.99-1.26) | 854 (314,031) | 0.99 (0.92-1.07) |
| *- Wholesale Trade* | 51 (36,871) | 0.88 (0.67-1.16) | 346 (138,117) | 0.93 (0.83-1.04) |
| *- Retail Trade* | 275 (118,327) | 1.17 (1.03-1.33) | 524 (184,282) | 1.04 (0.95-1.14) |
| Finance, Insurance and Real Estate (9) | 30 (9691) | 1.28 (0.89-1.83) | 51 (16,309) | 0.79 (0.60-1.04) |
| *- Finance* | <6 (1729) | -- | 6 (3388) | 0.43 (0.19-0.95) |
| *- Insurance Agencies and Real Estate* | 25 (7968) | 1.35 (0.91-2.00) | 45 (12,954) | 0.89 (0.66-1.20) |
| Community, Business and Personal Service (10) | 791 (396,744) | 0.98 (0.89-1.07) | 781 (271,399) | 1.03 (0.95-1.11) |
| *- Education and Related Services* | 158 (66,611) | 1.02 (0.87-1.21) | 206 (41,436) | 1.13 (0.99-1.31) |
| *- Health and Welfare Services* | 386 (168,105) | 1.05 (0.94-1.18) | 111 (31,701) | 0.98 (0.81-1.18) |
| *- Amusement and Recreation Services* | 9 (9999) | 0.71 (0.37-1.37) | 33 (13,221) | 1.06 (0.75-1.49) |
| *- Services to Business Management* | 62 (32,259) | 1.26 (0.98-1.63) | 177 (74,051) | 1.06 (0.91-1.23) |
| *- Personal Services* | 20 (7562) | 1.15 (0.74-1.78) | 20 (6507) | 0.98 (0.63-1.52) |
| *- Accommodation and Food Services* | 135 (97,781) | 0.85 (0.71-1.01) | 139 (73,287) | 0.91 (0.77-1.08) |
| *- Miscellaneous Services* | 51 (26,335) | 0.90 (0.68-1.19) | 116 (39,933) | 1.07 (0.89-1.29) |
| Public Administration and Defense (11) | 147 (75,093) | 0.93 (0.78-1.10) | 523 (127,816) | 1.01 (0.93-1.11) |
| *- Federal Administration* | 38 (15,635) | 0.99 (0.72-1.37) | 81 (18,665) | 0.88 (0.70-1.09) |
| *- Provincial Administration* | 39 (19,605) | 0.87 (0.64-1.20) | 118 (26,773) | 1.12 (0.93-1.34) |
| *- Local Administration* | 70 (39,133) | 0.98 (0.77-1.25) | 302 (78,489) | 1.04 (0.93-1.17) |
| *- Other Government Offices* | <6 (2328) | -- | 64 (9843) | 1.15 (0.90-1.47) |

a: Leukemia (excluding prevalent cases at cohort entry) is defined by the earliest date of diagnosis for any of the following SEER recode definitions: 35021, 35031, 35022, 35011, 35012, 35013, 35023, 35041, 35043.

b: Industry groups with case counts <6 for both sexes suppressed. c: Adjusted for age at start of follow-up and birth year.
SIC: Standard Industrial Classification; ODSS, Occupational Disease Surveillance System; HR, Hazard Ratio; CI: Confidence Interval

**Table S2.** Hazard ratios and 95% confidence intervals for leukemia subtypes, by division and major level industry groups.

| Industry Group (SIC Code) | Total Workers [b] | Acute Myeloid (n=1,888) [a] | | Chronic Myeloid (n=999) [a] | | Acute Lymphocytic (n=333) [a] | | Chronic Lymphocytic (n=3,119) [a] | |
|---|---|---|---|---|---|---|---|---|---|
| | | Cases | HR (95% CI) [c] | Cases | HR (95% CI) [c] | Cases | HR (95% CI) [c] | Cases | HR (95% CI) [c] |
| Agriculture (1) | 37,852 | 18 | 0.76 (0.48-1.21) | 11 | 0.86 (0.47-1.55) | 6 | 1.27 (0.57-2.86) | 37 | 0.96 (0.69-1.32) |
| Forestry, Fishing and Trapping (2/3) | 10,736 | 7 | 0.66 (0.31-1.38) | 7 | 1.21 (0.58-2.55) | <6 | -- | 22 | 1.15 (0.76-1.76) |
| *- Forestry* | 10,280 | 7 | 0.69 (0.33-1.45) | 7 | 1.27 (0.60-2.66) | <6 | -- | 22 | 1.20 (0.79-1.83) |
| Mines, Quarries, and Oil Wells (4) | 24,898 | 31 | 1.09 (0.76-1.56) | 21 | 1.39 (0.90-2.15) | 6 | 1.43 (0.64-3.23) | 77 | 1.48 (1.18-1.85) |
| *- Metal Mines* | 14,266 | 19 | 1.00 (0.63-1.57) | 19 | 1.92 (1.22-3.04) | <6 | -- | 56 | 1.58 (1.21-2.06) |
| *- Quarries and Sandpits* | 4226 | <6 | -- | <6 | -- | <6 | -- | 10 | 1.59 (0.86-2.97) |
| *- Services Incidental to Mining* | 4773 | <6 | -- | <6 | -- | <6 | -- | 10 | 1.40 (0.75-2.61) |
| Manufacturing Industries (5) | 716,893 | 623 | 0.96 (0.87-1.05) | 367 | 1.12 (0.98-1.28) | 112 | 0.99 (0.79-1.25) | 1084 | 0.99 (0.92-1.07) |
| *- Food and Beverage* | 93,181 | 81 | 1.03 (0.82-1.29) | 48 | 1.16 (0.87-1.56) | 19 | 1.38 (0.87-2.20) | 144 | 1.10 (0.93-1.30) |
| *- Rubber and Plastics Products* | 55,202 | 29 | 0.70 (0.48-1.01) | 19 | 0.85 (0.54-1.34) | <6 | -- | 58 | 0.87 (0.67-1.13) |
| *- Textile* | 19,766 | 16 | 0.93 (0.57-1.53) | 8 | 0.90 (0.45-1.81) | <6 | -- | 22 | 0.81 (0.53-1.23) |
| *- Clothing* | 10,299 | 10 | 0.95 (0.51-1.78) | <6 | -- | <6 | -- | 12 | 0.77 (0.44-1.36) |
| *- Wood* | 40,080 | 31 | 1.10 (0.77-1.57) | 11 | 0.70 (0.38-1.26) | <6 | -- | 34 | 0.70 (0.50-0.99) |
| *- Furniture and Fixtures* | 33,624 | 33 | 1.16 (0.82-1.64) | 20 | 1.31 (0.84-2.04) | <6 | -- | 37 | 0.76 (0.55-1.06) |
| *- Paper and Allied Industries* | 25,818 | 20 | 0.79 (0.51-1.23) | 13 | 0.97 (0.56-1.68) | <6 | -- | 47 | 1.07 (0.80-1.43) |
| *- Printing, Publishing, and Allied Industries* | 26,694 | 20 | 0.94 (0.60-1.46) | 10 | 0.89 (0.47-1.65) | <6 | -- | 45 | 1.31 (0.98-1.76) |
| *- Primary Metal Industries* | 42,737 | 43 | 0.89 (0.66-1.21) | 34 | 1.34 (0.95-1.89) | <6 | -- | 96 | 1.10 (0.89-1.34) |
| *- Metal Fabricating* | 132,775 | 104 | 0.98 (0.80-1.19) | 64 | 1.11 (0.86-1.44) | 20 | 1.03 (0.65-1.62) | 154 | 0.84 (0.71-0.98) |
| *- Machinery* | 6530 | <6 | -- | <6 | -- | <6 | -- | 17 | 1.26 (0.78-2.04) |
| *- Electrical Product* | 38,900 | 44 | 1.22 (0.90-1.64) | 10 | 0.53 (0.28-0.99) | <6 | -- | 47 | 0.79 (0.59-1.05) |
| *- Transportation Equipment* | 131,873 | 133 | 1.06 (0.89-1.26) | 88 | 1.33 (1.07-1.66) | 24 | 1.14 (0.75-1.73) | 280 | 1.30 (1.15-1.47) |
| *- Non-Metallic Mineral Products* | 25,787 | 21 | 0.89 (0.58-1.36) | 17 | 1.34 (0.83-2.16) | <6 | -- | 38 | 0.91 (0.66-1.25) |
| *- Chemical and Chemical Products* | 22,723 | 15 | 0.78 (0.47-1.29) | 7 | 0.69 (0.33-1.45) | <6 | -- | 26 | 0.81 (0.55-1.19) |
| *- Miscellaneous Manufacturing* | 57,809 | 41 | 0.79 (0.58-1.07) | 33 | 1.20 (0.85-1.70) | 9 | 1.01 (0.52-1.95) | 96 | 1.08 (0.88-1.33) |
| Construction (6) | 227,398 | 195 | 1.11 (0.96-1.30) | 85 | 0.86 (0.68-1.07) | 29 | 0.92 (0.62-1.36) | 315 | 1.01 (0.89-1.13) |
| *- General Contractors* | 76,924 | 66 | 0.99 (0.78-1.27) | 29 | 0.80 (0.55-1.16) | 8 | 0.71 (0.35-1.44) | 121 | 1.03 (0.85-1.23) |
| *- Special-Trade Contractors* | 159,640 | 136 | 1.16 (0.97-1.38) | 58 | 0.87 (0.67-1.14) | 21 | 0.99 (0.63-1.54) | 211 | 1.01 (0.88-1.16) |
| Transportation, Communication and Other Utilities (7) | 214,533 | 198 | 1.15 (0.99-1.34) | 113 | 1.24 (1.02-1.52) | 21 | 0.68 (0.44-1.06) | 331 | 1.12 (1.00-1.26) |
| *- Transportation* | 147,427 | 137 | 1.18 (0.99-1.41) | 73 | 1.17 (0.92-1.49) | 11 | 0.52 (0.29-0.96) | 214 | 1.06 (0.92-1.22) |
| *- Communication* | 34,278 | 24 | 0.82 (0.55-1.23) | 25 | 1.67 (1.12-2.48) | 6 | 1.23 (0.55-2.75) | 47 | 0.98 (0.73-1.30) |
| *- Electric, Power, Gas, and Water Utilities* | 24,101 | 34 | 1.53 (1.09-2.15) | 9 | 0.75 (0.39-1.44) | <6 | -- | 62 | 1.58 (1.23-2.04) |
| *- Storage* | 11,640 | 6 | 0.86 (0.38-1.91) | 8 | 2.09 (1.04-4.20) | <6 | -- | 16 | 1.37 (0.84-2.24) |
| Wholesale and Retail Trade (8) | 468,180 | 292 | 0.96 (0.84-1.09) | 167 | 1.04 (0.88-1.23) | 62 | 1.05 (0.79-1.39) | 504 | 1.05 (0.95-1.16) |
| *- Wholesale Trade* | 174,966 | 98 | 0.88 (0.72-1.08) | 50 | 0.82 (0.62-1.09) | 18 | 0.83 (0.52-1.35) | 178 | 0.98 (0.84-1.14) |
| *- Retail Trade* | 302,459 | 199 | 1.00 (0.87-1.16) | 121 | 1.18 (0.97-1.43) | 44 | 1.14 (0.83-1.57) | 335 | 1.09 (0.97-1.22) |
| Finance, Insurance and Real Estate (9) | 25,999 | 23 | 1.00 (0.66-1.50) | 12 | 1.03 (0.58-1.83) | <6 | -- | 31 | 0.80 (0.56-1.15) |
| *- Insurance Agencies and Real Estate* | 20,921 | 19 | 1.05 (0.67-1.64) | 10 | 1.10 (0.59-2.05) | <6 | -- | 30 | 0.99 (0.69-1.43) |
| Community, Business and Personal Service (10) | 667,741 | 444 | 1.05 (0.94-1.18) | 221 | 1.04 (0.89-1.22) | 86 | 1.08 (0.83-1.41) | 618 | 0.95 (0.86-1.04) |
| *- Education and Related Services* | 108,040 | 96 | 1.05 (0.85-1.29) | 32 | 0.71 (0.50-1.01) | 17 | 1.18 (0.72-1.93) | 170 | 1.20 (1.03-1.40) |
| *- Health and Welfare Services* | 199,796 | 140 | 1.01 (0.84-1.21) | 73 | 1.12 (0.87-1.45) | 27 | 1.05 (0.69-1.59) | 194 | 1.00 (0.86-1.17) |
| *- Amusement and Recreation Services* | 23,181 | 18 | 1.54 (0.97-2.46) | <6 | -- | <6 | -- | 15 | 0.82 (0.50-1.37) |
| *- Services to Business Management* | 106,302 | 59 | 1.03 (0.79-1.34) | 42 | 1.39 (1.02-1.90) | 14 | 1.22 (0.71-2.09) | 92 | 1.02 (0.83-1.26) |

| | | | | | | | | | |
|---|---|---|---|---|---|---|---|---|---|
| - *Personal Services* | 14,069 | 20 | 1.95 (1.26-3.03) | <6 | -- | <6 | -- | 11 | 0.70 (0.38-1.26) |
| - *Accommodation and Food Services* | 170,745 | 88 | 1.04 (0.84-1.30) | 47 | 1.07 (0.79-1.43) | 23 | 1.23 (0.80-1.89) | 76 | 0.60 (0.48-0.76) |
| - *Miscellaneous Services* | 66,253 | 43 | 0.97 (0.72-1.32) | 23 | 1.01 (0.67-1.53) | <6 | -- | 74 | 1.07 (0.85-1.35) |
| Public Administration and Defense (11) | 202,885 | 171 | 0.98 (0.84-1.15) | 74 | 0.80 (0.63-1.02) | 27 | 0.92 (0.62-1.36) | 306 | 1.05 (0.94-1.18) |
| - *Federal Administration* | 34,297 | 35 | 1.02 (0.73-1.43) | 13 | 0.75 (0.43-1.30) | <6 | -- | 48 | 0.84 (0.63-1.12) |
| - *Provincial Administration* | 46,376 | 36 | 0.91 (0.66-1.27) | 15 | 0.74 (0.44-1.23) | <6 | -- | 81 | 1.26 (1.01-1.57) |
| - *Local Administration* | 117,602 | 96 | 1.03 (0.84-1.26) | 43 | 0.87 (0.64-1.19) | 16 | 1.00 (0.61-1.66) | 167 | 1.07 (0.91-1.25) |
| - *Other Government Offices* | 12,171 | 17 | 1.07 (0.66-1.73) | <6 | -- | <6 | -- | 29 | 1.01 (0.70-1.46) |

a: Cases defined by the earliest date of diagnosis for each leukemia subtype, based on the following SEER recode definitions: Acute Myeloid (35021, 35031), Chronic Myeloid (35022), Acute Lymphocytic (35011), Chronic Lymphocytic (35012); excluding prevalent cases at cohort entry.
b: Industry groups with case counts <6 for all subtypes suppressed. c: Adjusted for age at start of follow-up, birth year, and sex.
SIC: Standard Industrial Classification; ODSS, Occupational Disease Surveillance System; HR, Hazard Ratio; CI: Confidence Interval
